# Obtaining of ZnO/Fe_2_O_3_ Thin Nanostructured Films by AACVD for Detection of ppb-Concentrations of NO_2_ as a Biomarker of Lung Infections

**DOI:** 10.3390/bios13040445

**Published:** 2023-03-31

**Authors:** Artem S. Mokrushin, Yulia M. Gorban, Aleksey A. Averin, Philipp Yu. Gorobtsov, Nikolay P. Simonenko, Yury Yu. Lebedinskii, Elizaveta P. Simonenko, Nikolay T. Kuznetsov

**Affiliations:** 1 Kurnakov Institute of General and Inorganic Chemistry of the Russian Academy of Sciences, Moscow 119991, Russia; julia_gorban@bk.ru (Y.M.G.); phigoros@gmail.com (P.Y.G.); n_simonenko@mail.ru (N.P.S.); ep_simonenko@mail.ru (E.P.S.); ntkuz@igic.ras.ru (N.T.K.); 2 Faculty of Technology of Inorganic Substances and High Temperature Materials, Mendeleev University of Chemical Technology of Russia, Moscow 125047, Russia; 3 Frumkin Institute of Physical Chemistry and Electrochemistry, Russian Academy of Sciences, Moscow 199071, Russia; alx.av@yandex.ru; 4 Moscow Institute of Physics and Technology, Dolgoprudny 141700, Russia; yylebedinskij@mephi.ru

**Keywords:** AACVD, chemoresistive gas sensors, zinc oxide (ZnO), iron oxide (Fe_2_O_3_), thin films, nanocomposites, nitrogen dioxide (NO_2_), two-dimensional nanomaterials

## Abstract

ZnO/Fe_2_O_3_ nanocomposites with different concentration and thickness of the Fe_2_O_3_ layer were obtained by two-stage aerosol vapor deposition (AACVD). It was shown that the ZnO particles have a wurtzite structure with an average size of 51–66 nm, and the iron oxide particles on the ZnO surface have a hematite structure and an average size of 23–28 nm. According to EDX data, the iron content in the films was found to be 1.3–5.8 at.%. The optical properties of the obtained films were studied, and the optical band gap was found to be 3.16–3.26 eV. Gas-sensitive properties at 150–300 °C were studied using a wide group of analyte gases: CO, NH_3_, H_2_, CH_4_, C_6_H_6_, ethanol, acetone, and NO_2_. A high response to 100 ppm acetone and ethanol at 225–300 °C and a high and selective response to 300–2000 ppb NO_2_ at 175 °C were established. The effect of humidity on the magnitude and shape of the signal obtained upon NO_2_ detection was studied.

## 1. Introduction

Semiconductor gas sensors based on metal oxides (MOS sensors) are widely used to detect toxic and explosive gases in air [1]. Devices with gas sensors of this type have a relatively low cost, high speed, high accuracy, high miniaturization potential, and are easy to operate [2].

Zinc oxide (*n*-type semiconductor with E_g_ = 3.37 eV) is one of the most widely used gas-sensitive materials in MOS sensors [3]. ZnO thin films exhibit good electrical conductivity, chemical stability, high ultraviolet (UV) absorption, and low toxicity [4,5]. Despite the unique physical and chemical properties, individual ZnO in the composition of chemoresistive gas sensors has some drawbacks: limited maximum sensitivity, low selectivity, high operating temperature, and long response and recovery times [6].

Various approaches to ZnO modification are used to improve its gas sensing properties. Increase in sensitivity can be achieved by using various transition metals (Fe, Cu, Ni, Y, etc.) as dopants [7,8,9,10]. Doping with lanthanides (Ce, Er) can not only improve the sensor response, but it also reduces the operating temperature [11,12]. Decoration with noble metals (Pd, Pt, Au, etc.) allows a significant increase in the response and selectivity to certain gases due to chemical and electronic sensitization [13,14,15,16]. Creating nanocomposites with other metal oxides (SnO_2_, CuO, NiO, etc.) is also an effective method to improve gas-sensitive properties by forming *p*-*n* or *n*-*n* heterojunctions and changing the thickness of the electron-depleted layer [17,18,19,20]. A suitable material for the synthesis of composites with a *n*-*n* heterojunction is α-Fe_2_O_3_ (a n-type semiconductor with a bandgap of 2.2 eV), which is readily accessible and relatively cheap [21,22].

A wide variety of synthetic techniques and approaches are used to produce ZnO/α-Fe_2_O_3_ nanocomposites. Thus, in [1] ZnO, nanorods were produced by chemical solution deposition (CSD) and then doped with α-Fe_2_O_3_ using liquid-phase ion layering (SILAR). This approach increased the response to ethanol (400 ppm) from 15.5 at 300 °C to 39 at 240 °C. In [23], zinc oxide was obtained by thermal evaporation followed by solvothermal precipitation of α-Fe_2_O_3_. The film had a seven-fold higher response to 2000 ppm ethanol compared to individual ZnO. In [24], the ZnO/α-Fe_2_O_3_ nanocomposite was obtained by solvothermal method. This material exhibited an increase in response to 100 ppm acetone from 7.2 to 29.9 at 290 °C, as well as a decrease in detection time compared to individual iron oxide. ZnO particles in the shape of three-dimensional tetrapods, doped with Fe_2_O_3_ nanoparticles, was obtained by flame transport synthesis, followed by addition of iron microparticles and annealing. The addition of Fe_2_O_3_ allowed us to achieve a high response to 100 ppm ethanol at room temperature and reduced the dependence of sensing properties on humidity [25]. In work [26], ZnO nanofilaments were obtained by the vapor–liquid–solid process, and then the precursor solution for α-Fe_2_O_3_ was deposited on ZnO by spin-coating, with resulting sample further annealed in a nitrogen atmosphere. At the Fe_2_O_3_ concentration of 0.05 M, the highest response value (18.8) to 100 ppm CO at 300 °C was achieved.

Compared to other synthesis techniques, aerosol-assisted chemical vapor deposition (AACVD) is a convenient way to produce thin nanostructured films of a given chemical composition with different morphologies. This method possesses a simple hardware design, low cost, and availability of precursors [27,28,29]. It is based on the atomization of a liquid precursor solution into aerosol droplets, which are subsequently transported to the heated reaction zone, where the solvent undergoes rapid evaporation and/or decomposition, forming a film of a given chemical composition on the substrate surface [30]. The literature contains data on the formation of ZnO/Fe_2_O_3_ nanocomposite with developed morphology by two-stage AACVD [31]. ZnCl_2_ solution in ethanol was used as a precursor to obtain ZnO particles at a synthesis temperature of 450 °C, then, at the same temperature, Fe_2_O_3_ was deposited on the obtained ZnO particles using acetone solution of FeCl_3_∙6H_2_O. This approach made it possible to obtain spear-shaped ZnO rods with a height of 1.2 μm and a diameter of 80 nm with flake-shaped Fe_2_O_3_ particles deposited on them. The total thickness of these structures formed on ZnO rods is about 200 nm.

Currently, gas sensors based on metal oxides are widely used not only in the environmental, automotive, and chemical industries for the detection of various gases, but they are also used in medicine. Methods of noninvasive diagnostics and patient monitoring based on the analysis of exhaled air using chemoresistive gas sensors are a modern alternative to more complex analytical methods, such as chromatography, mass spectrometry, etc. Such devices, based on MOS-gas sensors, are portable and easy to use, as well as allowing to diagnose diseases at an early stage [32]. Nevertheless, various studies are ongoing to develop the topology of such devices, as well as the search for new gas-sensitive materials capable of detecting gases that are biomarkers of various diseases [33,34]. Such disease biomarkers can be volatile organic compounds (VOC)—ethanol, acetone, toluene, as well as inorganic compounds (NO_x_, H_2_S, NH_3_) [35]. For example, increased concentrations of NO_x_ in exhaled air have been reported in patients with inflammatory lung diseases, such as bronchial asthma, bronchiectasis, and chronic obstructive pulmonary disease (COPD) [32,36,37]. The use of gas sensors in medicine requires the registration of analyte gases (NO_2_, ethanol, acetone, toluene, H_2_S, and others) at concentrations of less than 100 ppb, which is significantly below the lower detection limits for environmental monitoring (about tens and hundreds ppm) [38,39]. Thus, gas sensors used for medical purposes must be highly sensitive to ultra-low gas concentrations, selective, and have minimal dependence on humidity.

We found no data on the use of the AACVD process to produce ZnO/Fe_2_O_3_ nanocomposites for chemical gas sensing, so the aim of this work was to obtain thin nanostructured ZnO/Fe_2_O_3_ films by the AACVD method for chemoresistive gas sensors, as well as to study the influence of the Fe_2_O_3_ amount on the gas-sensitive properties, including the detection of NO_2_, a biomarker of various diseases.

## 2. Materials and Methods

### 2.1. AACVD Synthesis of ZnO/Fe_2_O_3_ Films

**Figure 1:**
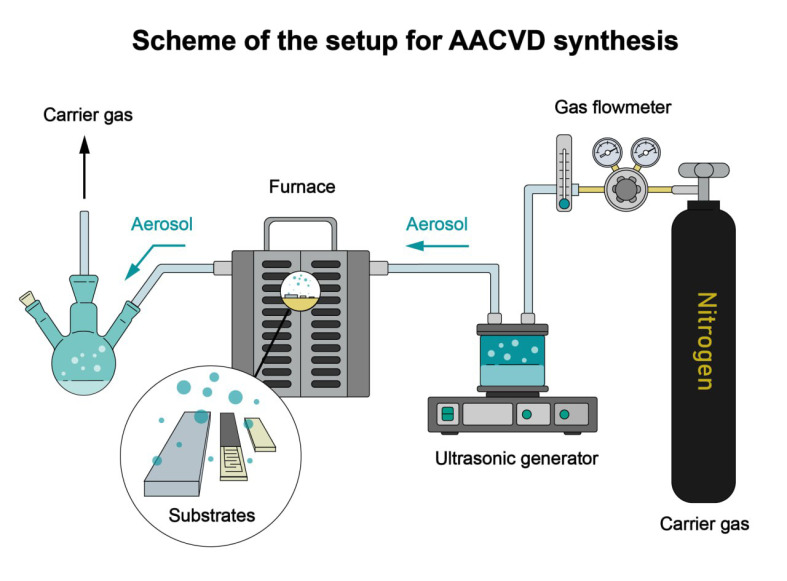
Scheme of the setup for AACVD synthesis of ZnO/Fe_2_O_3_ thin films.

An amount of 0.05 M solutions of zinc acetylacetonate [Zn(H_2_O)(O_2_C_5_H_7_)_2_] and iron acetylacetonate [Fe(O_2_C_5_H_7_)_3_] in methanol CH_3_OH (>99%, Chimmed) were used in the AACVD synthesis. Zinc and iron acetylacetonates were synthesized using zinc nitrate Zn(NO_3_)_2_ 6H_2_O (>98%, “Reachem”), iron chloride, FeCl_3_, 6H_2_O (>98%, “Reachem”), and acetylacetone (>98%, “Ecos-1”) by neutralization of their solutions with 5% ammonia hydrate solution (NH3⋅H2O, >99%, “Ecos-1”). ZnO films were obtained using a special laboratory setup consisting of a gas flow meter, an ultrasonic generator (Albedo IN-7), a furnace with a flow chamber, and an aerosol trapping system. To maintain constant conditions of aerosol formation, flow-through water cooling of the ultrasonic generator was performed. Aerosol deposition was performed in a flow furnace chamber on various substrates (glass, aluminum oxide, and an Al_2_O_3_ sensor with platinum counter-pin electrodes and a microheater on the reverse side [40]) at a decomposition temperature of 400 °C. Nitrogen (high purity grade “6.0”) was used as a carrier gas, with a flow rate of 300 mL/min. At the first stage, using zinc acetylacetonate solution, ZnO film was obtained (sample Z) with a deposition time of 60 min. In the second step, a Fe_2_O_3_ film was deposited on the surface of the ZnO film using iron acetylacetonate solution for 10, 20, and 30 min at 400 °C, which are called Z1Fe, Z2Fe, and Z3Fe, respectively, in the further text. For additional analysis by Raman spectroscopy, a film of individual Fe_2_O_3_ was synthesized on an Al_2_O_3_ substrate under the following conditions: sputtering time of 45 min at 400 °C. The scheme of the laboratory setup used for AACVD synthesis of ZnO/Fe_2_O_3_ thin nanostructured films used in this work is shown in Figure 1. After synthesis, additional heat treatment of the coatings was performed at 350 °C for 2 h in the air for additional removal of organic compounds from the surface of the films.

### 2.2. Instrumentation

XRD patterns of the coatings on glass substrates were recorded on a D8 Advance X-ray diffractometer (Bruker) with CuKα emission in the range of 2θ 25°–65° in 0.02° increments with signal accumulation at the point for 1.8 s. The morphology and microstructure of the films on Al_2_O_3_ substrates were studied using a NVision 40 three-beam workstation (Carl Zeiss). Electronic UV transmission spectra of ZnO/Fe_2_O_3_ films on glass substrates were recorded using a SF-56 UV-Vis spectrophotometer. Raman spectra were obtained with a Renishaw inVia Reflex spectrometer using a 405 nm diode laser as the excitation source. All spectra were recorded in the 100–2000 cm^−1^ range with a spectral resolution of ~3 cm^−1^ through a 50× (NA 0.5, FN 26.5) magnification lens, with an irradiated site diameter of ~2 μm. The incident radiation power was less than 2.5 mW. Grating: 2400, and the signal accumulation time was 300 s.

The gas-sensitive properties were measured on a specialized precision setup [41,42,43]. The gas environment in the quartz cell was created using four Bronkhorst gas flow controllers with maximum flow rates of 1, 50, 100, and 200 mL/min. The temperature of the sensor element was regulated using a built-in platinum microheater pre-calibrated using a Testo 868 thermal imager. The resulting film was studied for sensitivity to the following analyte gases: CO, H_2_, CH_4_, NH_3_, benzene, acetone, ethanol, and NO_2_. The corresponding test gas mixtures in air were used as a source of the analyzed gases, and synthetic air was used to construct a baseline. To measure the signal at different relative humidity (RH), we used a special unit with a bubbler, and the RH of the gas mixture was controlled by a digital flow-through hygrometer «Excis». Electrical resistance of oxide films was measured using a Fluke 8846A (6.5 Digit Precision Multimeter) with an upper limit of 1 GOhm. The response to CO, H_2_, CH_4_, NH_3_, benzene, acetone, and ethanol was calculated using the formula:

S = R_Air_/R, (1)

where R_Air_ is the resistance of the oxide film in the synthetic air medium; R—in the environment with a given concentration of the analyte gas. The response to NO_2_ was calculated by the inverse relation (1).

The selectivity coefficient was calculated by the following equation:

Sel = S_NO2_/S_2_, (2)

where S_NO2_ is the response when detecting 2 ppm NO_2_; S_2_ is the response to a given gas concentration (in our case it was 100 ppm ethanol or acetone), to which the highest response value after NO_2_ was observed.

The following ratio was used to calculate «response drop» (RD) in a humid atmosphere $RD=\frac{R_{0}-R_{RH}}{R_{0}}\times 100\%$, where $R_{0}$ is the response at 0% RH, $R_{RH}$ is the response at the corresponding RH.

## 3. Results and Discussion

### 3.1. Chemical and Phase Composition

**Figure 2:**
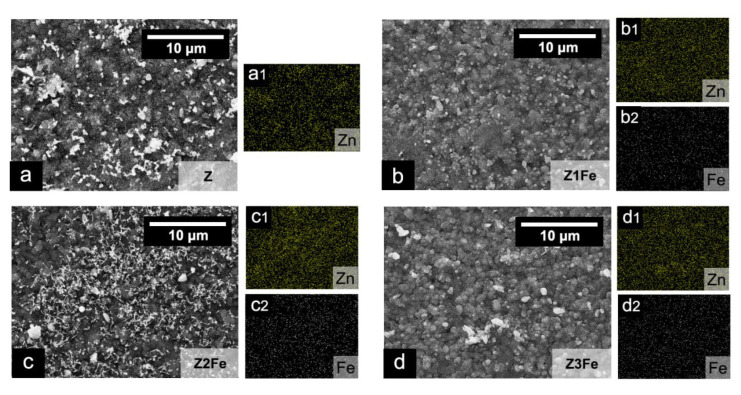
EDX mapping of zinc and iron in thin films of ZnO/Fe_2_O_3_: (**a**) Z, (**b**) Z1Fe, (**c**) Z2Fe, and (**d**) Z3Fe with Zn and Fe element distribution (**a1**–**d2**).

The chemical composition of the obtained ZnO/Fe_2_O_3_ films was studied by energy dispersive X-ray spectroscopy (EDX). The EDX mapping of zinc and iron shows (Figure 2) that both metals are evenly distributed on the surface of the obtained films. The calculated EDX values showed that the iron content in Z1Fe, Z2Fe and Z3Fe films was 1.3, 4.8 and 5.8 at.%, respectively. Thus, when the sputtering time of the Fe_2_O_3_ film increases from 10 to 30 min, the iron content of the ZnO/Fe_2_O_3_ nanocomposite films increases proportionally.

**Figure 3:**
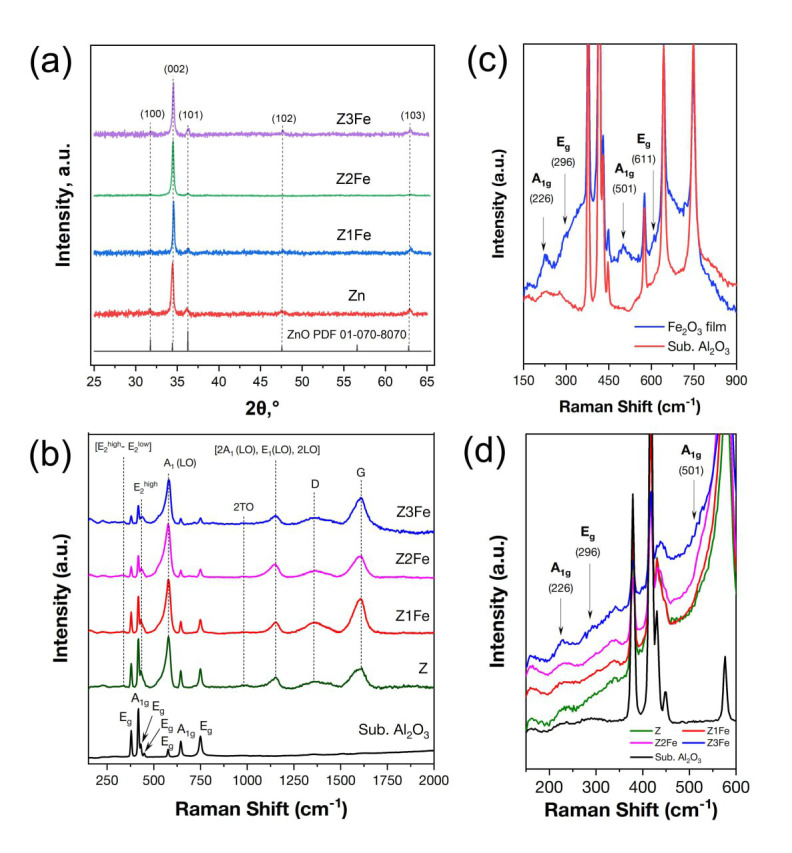
XRD-pattern for ZnO/Fe_2_O_3_ films on glass substrate (**a**) and Raman spectra for ZnO/Fe_2_O_3_ thin films on Al_2_O_3_ (**b**); enlarged Raman spectra of individual Fe_2_O_3_ (**c**) and ZnO/Fe_2_O_3_ films on Al_2_O_3_ substrate (**d**).

Due to the small thickness of the synthesized films, the diffractograms obtained for films on polycrystalline Al_2_O_3_ substrates are uninformative. Against the background of intense substrate reflexes, low-intensity film reflexes are practically indiscernible. Therefore, X-ray phase analysis of ZnO/Fe_2_O_3_ thin films was performed on X-ray amorphous glass substrates. According to the data of X-ray phase analysis (Figure 3a) of ZnO/Fe_2_O_3_ thin films on glass substrates it was found that, for all samples, the presence of characteristic reflexes of hexagonal ZnO wurtzite phase (spatial group P63mc, PDF 01-070-8070) is observed [44]. The most intense reflex (002) is observed at 34.4° 2θ, which indicates a strong preferential orientation in the [001] direction. Crystallite growth variations are related to solvent polarity, so the intensity of reflex (002) may be due to the use of methanol, which has one of the highest polarities of all organic solvents, in the AACVD process [30]. The mean sizes of ZnO crystallites calculated by the Scherrer formula for samples Z, Z1Fe, Z2Fe, and Z3Fe were 40, 42, 30, and 35 nm, respectively. No separate iron-containing phases were detected on the diffractograms, which may be due to the low concentration of iron oxide, the small thickness of the Fe_2_O_3_ layer, and the instrumental feature of the diffractometer in which the copper tube was installed. It is also worth noting that, for samples Z1Fe, Z2Fe, and Z3Fe, no shift in reflexes relative to individual ZnO is observed, which is an indirect sign of the formation of a two-phase nanocomposite rather than a solid solution.

Additionally, synthesized ZnO/Fe_2_O_3_ thin films on Al_2_O_3_ substrates were studied by Raman spectroscopy. As can be seen from Figure 3b, besides the peaks related to the α-Al_2_O_3_ substrate (A1g at 418 and 644 cm^−1^ and Eg at 378, 430, 450, 576 and 749 cm^−1^) [45], an intense ZnO peak at 577 cm^−1^, corresponding to A1(LO) mode, is observed in all samples. In addition, peaks assigned to the (E_2_^high^–E_2_^low^), E_2_^high^, 2TO, and [2A1 (LO), E1(LO), 2LO] modes were observed at 332, 438, 982, and 1153 cm^−1^, respectively [46]. Two peaks at 1356 and 1607 cm^−1^ can be attributed to the D- and G-bands of residual carbon that remained after the synthesis. Figure 3c shows the spectrum of the individual iron oxide film. As can be seen, besides the α-Al_2_O_3_ substrate peaks, bands at 226, 501 (A_1g_) and 296 (E_g_) cm^−1^ with low intensity, characteristic of α-Fe_2_O_3_ (trigonal syngony, hematite phase, space group R3c), are observed [47]. Figure 3d shows that the α-Fe_2_O_3_ peaks at 226 and 296 cm^−1^ in the spectra of Z1Fe, Z2Fe, and Z3Fe samples are also low in intensity. The weak signal from α-Fe_2_O_3_ may indicate the low concentration and thickness of the iron oxide layer. Slightly more intense peaks are observed for the Z3Fe sample due to the higher concentration of iron oxide. The obtained peaks related to Fe_2_O_3_, although low-intensity, allow us to establish the formation of the ZnO/α-Fe_2_O_3_ nanocomposite.

**Figure 4:**
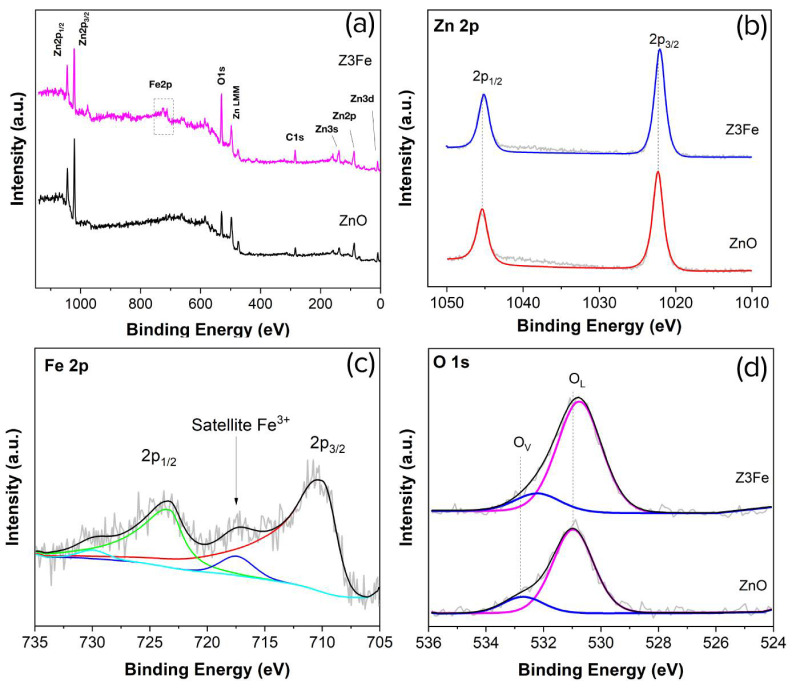
XPS spectra of ZnO and Z3Fe thin films: overview (**a**), Zn 2p (**b**), Fe 2p (**c**), and O 1s (**d**); on the spectra (**c**,**d**), coloured lines indicate the different components.

Figure 4a shows an overview XPS spectrum of Z and Z3Fe samples. As can be seen, the spectrum contains signals of the element peaks: Zn, O, and C (for the sample ZnO), as well as weakly intense signals from Fe (for the sample Z3Fe). It is likely that carbon is in the composition of CO_2_ sorbed on the surface of the materials, as well as residual products after the synthesis. Figure 4b shows the XPS spectra of Zn 2p. Two peaks of Zn 2p_1/2_ and Zn 2p_3/2_ can be seen on the plot: at 1045.4 and 1022.3 eV, 1045.2 and 1022.1 eV for ZnO, and Z3Fe samples, respectively. The spin-orbit splitting was 23.1 eV, which is typical for ZnO with the wurtzite structure and agrees well with the literature data [48,49].

Figure 4c shows the XPS spectra of the Fe 2p sample Z3Fe. Two pronounced peaks of Fe 2p_1/2_ and Fe 2p_3/2_ at 723.5 and 710.3 eV can be seen on the plot. The signals obtained can equally be attributed to both Fe_2_O_3_ and Fe_3_O_4_ [50]. The main difference between Fe_2_O_3_ and Fe_3_O_4_ on the XPS spectra is the presence of a satellite peak for Fe_2_O_3_ between Fe 2p_1/2_ and Fe 2p_3/2_ [51,52], which was also observed in our case at 717.5 eV. Therefore, based on the XPS data obtained, we can assert the formation of the Fe_2_O_3_ phase within the thin films, which agrees well with the Raman spectroscopy data.

Figure 4d shows the O 1s spectra. As can be seen, 2 O_L_ and O_V_ peaks are present in the spectra: at 531.0 and 532.8 eV, as well as 530.8 and 532.3 eV, for ZnO and Z3Fe samples, respectively. The O_L_ peak refers to the lattice oxygen (O^2−^) in the crystal structure of ZnO and Fe_2_O_3_, while the O_V_ signal probably refers to oxygen vacancies and/or oxygen-containing groups (e.g., O^−^) sorbed on the surface [53,54]. When Fe_2_O_3_ is modified, the maxima of the main peaks of all elements are shifted by 0.2–0.5 eV, which may be related to the effect of the modifying additive and the formation of the Schottky barrier in the nanocomposite.

### 3.2. Microstructure and Morphology

**Figure 5:**
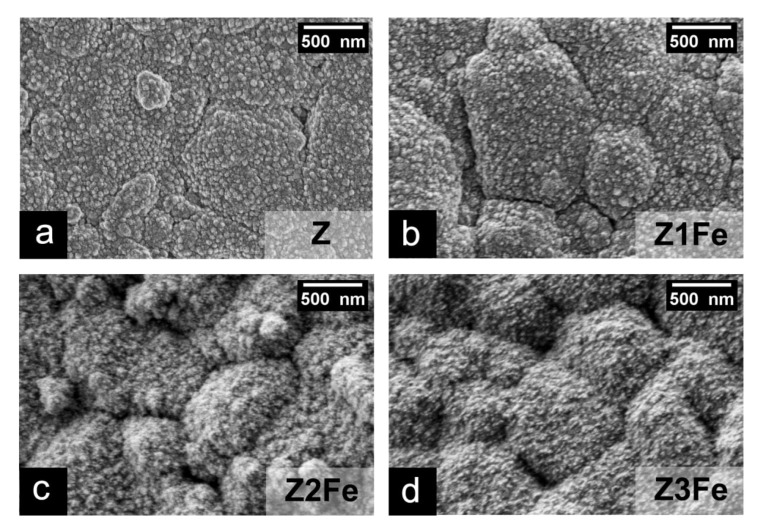
SEM micrographs for thin films of ZnO/Fe_2_O_3_: Z (**a**), Z1Fe (**b**), Z2Fe (**c**), and Z3Fe (**d**).

The microstructure and dispersity of ZnO/Fe_2_O_3_ thin films on Al_2_O_3_ substrates were studied by scanning electron microscopy (SEM). As can be seen (Figure 5), all films uniformly cover the substrate surface, forming a continuous coating. The lower layer of ZnO in all films consists of spherical nanoparticles. In sample Z, the size of ZnO nanoparticles is 66 ± 13 nm, Z1Fe: 50 ± 14 nm, and Z2Fe: 51 ± 10 nm, and, in Z3Fe, the iron oxide covers the ZnO particles tightly, due to which it is not possible to calculate their size. The data obtained correlate well with the calculated crystallite values estimated using the Scherrer method on the obtained X-ray diffraction patterns. Z2Fe and Z3Fe samples show Fe_2_O_3_ nanoparticles on the ZnO surface, their size in the Z2Fe sample being 23 ± 4 nm, and, with increasing sputtering time (Z3Fe), their size increases to 28 ± 5 nm. Due to the low concentration of iron in the Z1Fe sample, it is not possible to estimate the size of Fe_2_O_3_ nanoparticles. 

**Figure 6:**
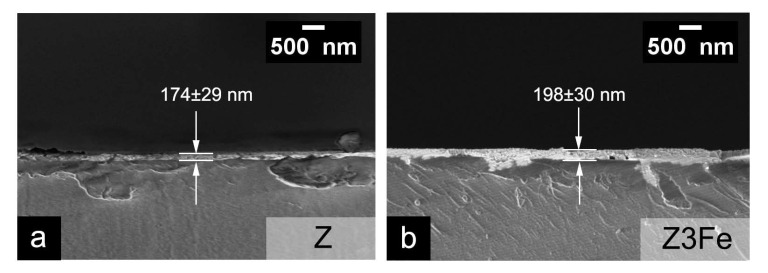
SEM microphotographs of chips of Z (**a**) and Z3Fe (**b**) samples on glass substrates.

Figure 6 shows microphotographs of chips of glass substrates with Z(a) and Z3Fe (b) films applied. The calculated film thickness values for Z, Z1Fe, Z2FE, and Z3Fe were 174 ± 29, 181 ± 11, 185 ± 40, and 198 ± 30 nm, respectively Thus, the thickness of the thin film increases with increasing Fe2O3 sputtering time.

Considering all of the above data (EDX, SEM, XRD, Raman, XPS) on the phase composition, microstructure, dispersity, and thickness of the obtained ZnO/Fe_2_O_3_ films, the following conclusions can be made. At the minimum deposition time of Fe_2_O_3_ (10 min, sample Z1Fe), Fe_2_O_3_ coating is formed on the surface of ZnO, consisting of individual clusters of nanoparticles, which seemingly do not constitute a continuous coating. When the Fe_2_O_3_ deposition time is increased up to 20–30 min (samples Z2Fe and Z3Fe), a thin continuous Fe_2_O_3_ coating with a thickness of about 10–20 nm with an average nanoparticle size of 23–28 nm begins to form on the ZnO surface. Thus, by varying the time of deposition of the modifying layer, it is possible to change the continuity and thickness of the obtained coating. Table 1 summarizes the properties of the obtained ZnO/Fe_2_O_3_ nanostructured thin films.

### 3.3. UV Transmittance Spectra and Optical Band Gap

**Figure 7:**
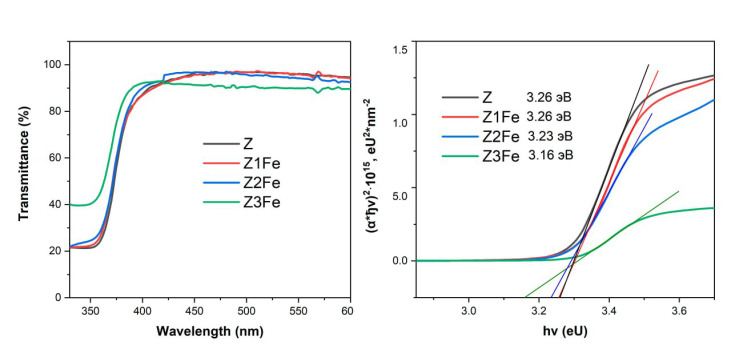
UV transmittance spectra (**a**) and Tauc plot (**b**) for thin films of ZnO/Fe_2_O_3_.

Figure 7a shows spectra of UV and visible transmittance of the films on glass substrates. It can be seen from the data that all films have a high transmittance in the visible range. The transmittance of Z and Z1Fe films ranges from 87 to 97% of visible light, Z1Fe—90–97%, and Z3Fe has the lowest transmittance in the visible range (90–92%), probably due to the dense coating of ZnO surface with Fe_2_O_3_ nanoparticles. The edge of the fundamental absorption band of the films corresponds to the transition of the electron from the valence band to the conduction band, and it can be used to calculate the optical band gap width. The optical band gap width was determined by plotting the Tauc plot (Figure 7b) [55]. The Eg values of ZnO/Fe_2_O_3_ films calculated by this method are 3.26, 3.26, 3.23, and 3.16 eV for samples Z, Z1Fe, Z2Fe, and Z3Fe, respectively. The narrowing of the band gap width can be explained by the strength of ionic bonding between metal ions and oxygen ions. The electronegativity values of Zn, Fe, and O are 1.5, 1.8, and 3.5, respectively [56], so the difference in energy levels between the s-state cation and the O 2p state in α-Fe_2_O_3_ is smaller than in ZnO. This testifies to the fact that the red shift of the absorption edge occurs mainly due to the formation of Fe s-levels below the conduction zone of ZnO [57].

### 3.4. Gas-Sensing Chemoresistive Properties

#### 3.4.1. Selectivity

**Figure 8:**
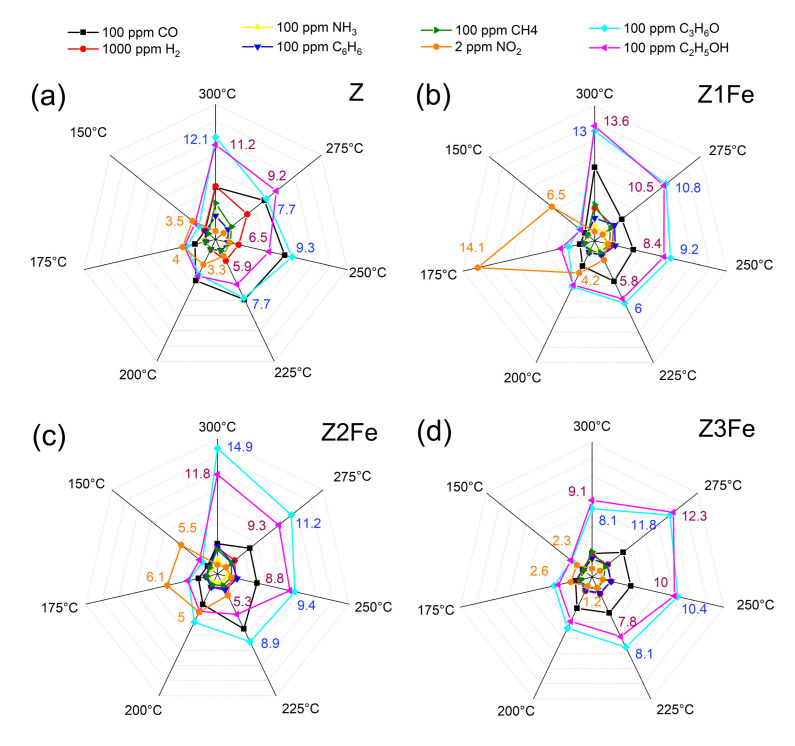
Selectivity diagrams responses to various gases (H_2_, CH_4_, CO, C_6_H_6_, NH_3_, ethanol, acetone, and NO_2_) at 150–300 °C of the samples: (**a**) Z, (**b**) Z1Fe, (**c**) Z2Fe, and (**d**) Z3Fe.

The obtained ZnO/Fe_2_O_3_ films showed good electrical conductivity, which allowed us to study chemoresistance responses to various gases (H_2_, CH_4_, CO, C_6_H_6_, NH_3_, ethanol, acetone, and NO_2_) in a wide temperature range: from 150 to 300 °C (Figure 8).

At lower operating temperatures, the resistance of the nanocomposite films was high enough that it was not possible to evaluate the gas sensitivity on the equipment used in the present work (electrical resistance measurements were limited to 1 GOhm). At elevated temperatures, the greatest response for all analyzed films was observed for VOCs: ethanol and acetone. The greatest sensitivity to the above gases was observed at 300 °C, with response values being: 11.2, 13.6, and 11.8 (100 ppm ethanol), as well as 12.1, 13.0, and 14.9 (100 ppm acetone) for samples Z, Z1Fe, and Z2Fe, respectively. For the sample with the highest iron content (Z3Fe), the maximum response (11.8 and 12.3 for ethanol and acetone, respectively) was observed at 275 °C. The response to other gases at 275–300 °C did not exceed 7.4, 8.7, 4.9, and 4.7 for Z, Z1Fe, Z2Fe, and Z3Fe, respectively. Upon lowering the operating temperature to 150 °C, a significant decrease in the responses to ethanol and acetone (to 2.1–3.2) was observed. When detecting NO_2_, a different dependence was observed: when the operating temperature was changed from 300 to 150 °C, the response significantly increased. Such phenomena are quite typical for ZnO-based receptor nanomaterials, which are characterized by increased sensitivity to various VOCs at elevated temperatures and to NO_2_ at moderate ones [58,59].

**Figure 9:**
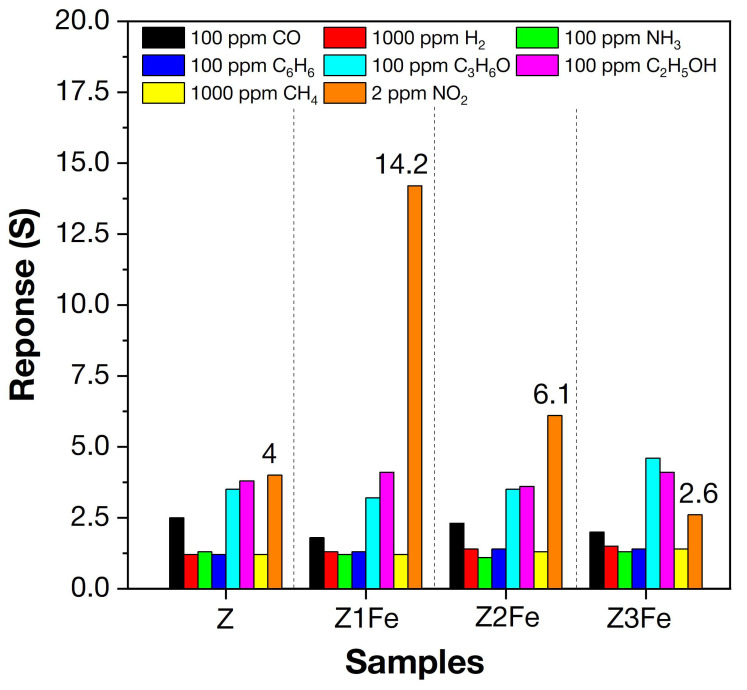
Selectivity histogram of the samples to various gases (H_2_, CH_4_, CO, C_6_H_6_, NH_3_, ethanol, acetone, and NO_2_) at 175 °C.

To select the optimal temperature for the detection of the analyte gas, several factors should be taken into account, among which the main ones are, definitely, selectivity and energy efficiency, which are determined by the operating temperatures at which the detection is performed. When analyzing the obtained array of responses of ZnO/Fe_2_O_3_ thin films to various gases at 150–300 °C, a low working temperature of 175 °C was chosen, and pronounced sensitivity to ultra-low NO_2_ concentrations was observed at this point. Next, the chemoresistive gas-sensitive properties upon NO_2_ detection at 175 °C were studied in more detail (Figure 9).

All samples of ZnO/Fe_2_O_3_ thin films showed unusually high sensitivity to NO_2_. An increase in electrical resistance (which is typical for n-type MOS) was observed when detecting NO_2_. When the concentration of NO_2_ increases above 2 ppm, the resistance upon response recording exceeds 1 GOhm, which does not allow its detection on the equipment used. Therefore, all further measurements upon NO_2_ detection were limited to the concentration of 2 ppm. The sample (Z1Fe) with the lowest iron content had the highest response (S = 14.2) and selectivity (Sel = 3.4) when detecting 2 ppm NO_2_. For other samples, the responses to 2 ppm NO_2_ are 4.0, 6.1, and 2.6, and the selectivity coefficients were 1.2, 1.7, and 0.6 for Z, Z2Fe, and Z3Fe, respectively. Thus, the sample (Z1Fe) with the lowest iron content has the highest selectivity at 2 ppm NO_2_. It should be noted that the comparison of responses to NO_2_ with other gases was carried out at their various concentrations, which differ by 50 times or more (100 ppm vs. 2 ppm NO_2_). Therefore, the obtained value (Sel = 3.4) for Z1Fe, as well as for other samples, is an extremely high value, showing good selectivity for NO_2_ detection.

**Figure 10:**
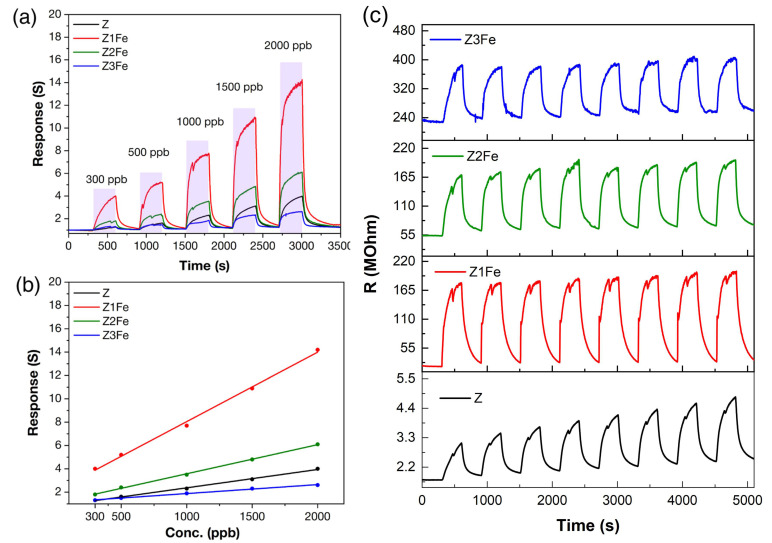
Sensitivity to 300–2000 ppb NO_2_ (**a**); dependences of response from NO_2_ concentration (**b**) and reproducibility of the signal when detecting 500 ppb NO_2_ (**c**) at 175 °C operating temperature and 0%RH for ZnO/Fe_2_O_3_ thin films.

Figure 10a shows the experimental data on the sensitivity to different NO_2_ concentrations of Z, Z1Fe, Z2Fe, and Z3Fe samples. The zinc oxide-based films show a high response in a wide range of ultra-low NO_2_ concentrations (300–2000 ppb), covering the threshold limit value (TLV) recommended by the National Institute for Occupational Safety and Health [60]. The response increases from 1.3 to 4.0, 4.0–14.2, 1.9–6.1, and 1.3–2.6 with increasing NO_2_ concentrations from 300 to 2000 ppb for Z, Z1Fe, Z2Fe, and Z3Fe, respectively.

Figure 10b shows the dependences of the response value on the concentration of NO_2_ in the gas mixture. The highest response over the entire NO_2_ concentration range was observed for the Z1Fe sample. All samples can be described well by a linear function. This dependence is typical for many chemoresistive gas sensors and correlates well with the available literature data [1,61]. Figure 10c shows the reproducibility of the signal when detecting 500 ppb NO_2_ at 0%RH. It can be seen that, for individual ZnO, there is a drift in the values. With the addition of iron, the signal becomes more stable with minimal drift of the resistance values.

The kinetic characteristics were evaluated: the response time changes nonlinearly with increasing analyte concentration, and, for concentrations of 300–2000 ppb, these values amount to 143–180, 118–203, and 121–160, as well as 58–176 s for Z, Z1Fe, Z2Fe, and Z3Fe, respectively.

**Figure 11:**
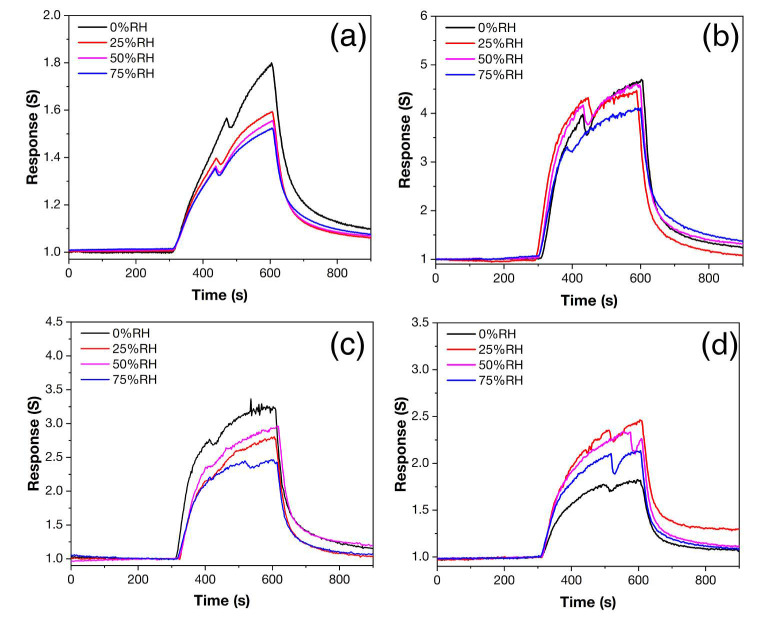
Response to 500 ppb NO_2_ at 175 °C and 0–75% RH of ZnO/Fe_2_O_3_ thin films: Z (**a**), Z1Fe (**b**), Z2Fe (**c**), and Z3Fe (**d**).

Figure 11 shows the responses of samples Z, Z1Fe, Z2Fe, and Z3Fe when detecting 500 ppb NO_2_ at different relative humidity values (RH). For individual ZnO, when the RH increases from 0 to 75%, a consistent decrease in the response value from 1.8 to 1.5 is observed (Figure 11a). The shape of the signal has a sharply pointed appearance, which may indicate that the equilibrium on the surface has not been established, which is due to the long process of establishing a stable signal. When doped with iron oxide, the shape of the responses becomes close to rectangular. For the sample Z1Fe (Figure 11b), the smallest influence of humidity on the signal is observed. At RH in the 0–75% range, the response decreases linearly from 4.6 to 4.1. The Z2Fe sample (Figure 11c) shows a response of 3.2–2.4 with a general trend of decreasing response with increasing humidity, but the signal at 50% RH is slightly greater than at 25% RH. A different behavior is exhibited by Z3Fe (Figure 8d), for which the response in a humid environment (2.3–2.5) is higher than in a dry atmosphere (1.8).

**Figure 12:**
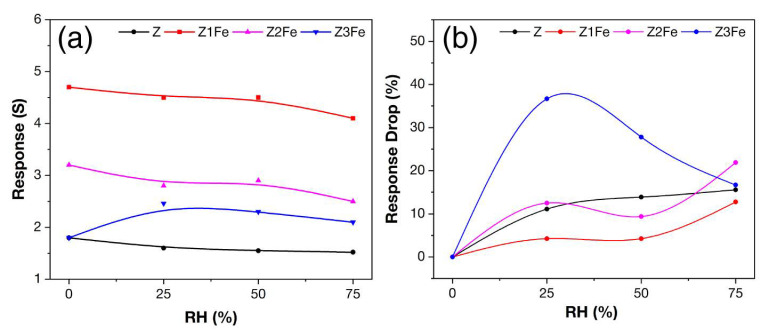
Dependence of response value on humidity (**a**) and response drop (RD) in a humid environment (**b**) when detecting 500 ppb NO_2_ at 175 °C with ZnO/Fe_2_O_3_ thin films.

Figure 12a summarizes the data on the effect of humidity on the response value of the obtained thin films. For samples Z, Z1Fe, and Z2Fe, the response dependence on RH can be described by the curve with a negative slope. The sample Z3Fe showed an increase in the response at 25% RH, and then a consistent decrease in its value was observed. Figure 12b shows the modulus of change in the response value relative to its value in dry air. As can be seen, the sample Z1Fe is the least affected by humidity among the samples, and the sample Z3Fe has the maximum sensitivity to humidity.

#### 3.4.2. Detection Mechanism

The resistive response in the detection of NO_2_ by individual ZnO results from various reactions, which can be described using the model for n-type semiconductors generally accepted in the literature. In an air environment at elevated temperatures, oxygen molecules adsorb onto the semiconductor surface, which leads to a change in the resistance of the material. Electrons from the conduction zone reduce O_2_ to ionic form (at 175 °C, O^−^ particles would be predominant) [62]. The presence of such ions on the ZnO surface contributes to the formation of the electronic structure of the core–shell type. The core is the inner region of the semiconductor particle, and the shell is the electron depletion layer (EDL) formed as a result of electron consumption during the reduction of O_2_ to O^−^.

In the work by Mei Chen et al. [63], the process of interaction of NO_2_ with zinc oxide was studied using a number of methods of in situ physicochemical analysis. The authors were able to show that, upon the intake of NO_2_, nitrites (NO_2_^−^) and then nitrates (NO_3_^−^) are formed in the first minutes with the participation of various defects in the crystal lattice of ZnO. The formation of NO_2_^−^_(ads)_ and NO_3_^−^_(ads)_ can also occur with the participation of electrons from the conduction band of ZnO, the lattice oxygen O_(lat)_ or the sorbed O^−^_(ads)_ ion [64]. In our previous work [59], while studying the thick zinc oxide films obtained by solvothermal method, we correlated the Mei Chen data with the results we obtained in the study of chemoresistive responses in NO_2_ detection and summarized them. More details on the mechanisms of NO_2_ detection by individual ZnO can be found in our work at the above reference.

Thus, based on the above data, we can conclude that the maximum response to NO_2_ of an individual ZnO thin film at 175 °C can be explained by the maximum intensity of NO_2_ adsorption in this temperature range. It is worth noting that, just as in our previous work, where a thick ZnO film was obtained by the solvothermal method, so in the present work, where a thin ZnO film was obtained by the AACVD method, zinc acetylacetonate was used as a precursor. In both cases, extremely NO_2_-sensitive receptor nanomaterials, based on ZnO, were obtained using completely different methods. Therefore, the choice of the precursor used probably also plays an important role in obtaining a highly sensitive receptor nanomaterial for chemoresistive gas sensors.

**Figure 13:**
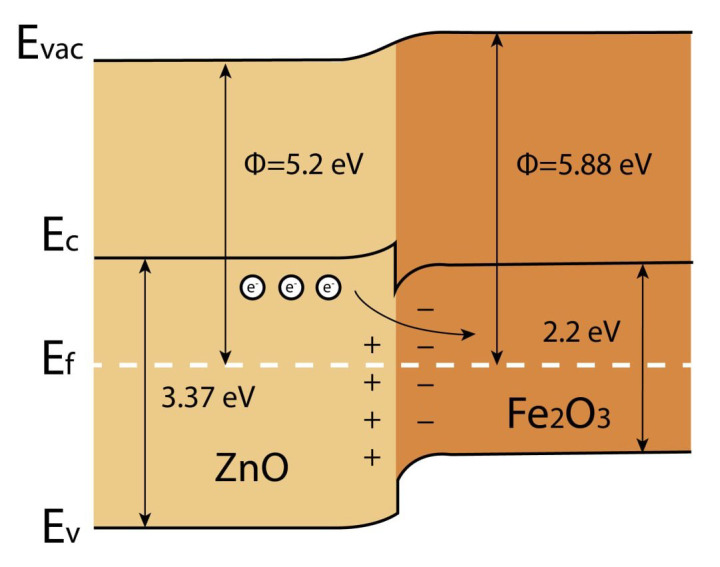
Energy diagram of heterojunction formation between ZnO and Fe_2_O_3_ in ZnO/Fe_2_O_3_ thin films.

The highest response over the entire NO_2_ concentration range for the Z1Fe sample can be attributed to the formation of a heterojunction (Figure 13). The Fermi level of Fe_2_O_3_ is lower than that of ZnO, so there is directional electron transfer from the conduction zone of ZnO to Fe_2_O_3_ until level alignment is achieved. Thus, an electron-depleted layer is formed at the boundary of the two semiconductors, which leads to significant chemical and electronic sensitization. In addition, the Fe_2_O_3_ nanoparticles can interact with NO_2_, which leads to the electron transfer from ZnO to Fe_2_O_3_ and widening of the electron-depleted ZnO layer [65]. The obtained cluster structure of Fe_2_O_3_ nanoparticles on the ZnO surface (sample Z1Fe) is suitable for the realization of the heterojunction, as described above, allowing us to improve the chemoresistive properties. As the thickness of the Fe_2_O_3_ layer increases, the sensitivity to NO_2_ decreases as the area covered by the Fe_2_O_3_ nanoparticles increases. Zinc oxide stops being the main gas-sensitive material, and the heterojunction effect ceases to be the limiting one.

In humid atmosphere, water molecules sorbed on the ZnO surface additionally participate in the detection mechanism. In many cases, humidity has a negative effect on the chemoresistive response and remains one of the main problems in gas sensing. After sorption, the H_2_O(ads) molecules react with point defects (zinc Zn(lat) and oxygen O(lat) in the crystal lattice, resulting in the formation of hydroxyl groups on the ZnO surface [3,66]. The released electrons enter the conduction band of ZnO, which leads to decrease in electrical resistance. The formed hydroxyl groups occupy the active adsorption centers on the ZnO surface, thus reducing the adsorption of NO_2_(ads) and O^−^(ads) and the intensity of the reactions, which contribute to a chemoresistive response.

For the sample Z1Fe with the Fe_2_O_3_ cluster structure, moisture has almost no effect on the response value. Additionally, with further increase in the thickness of the Fe_2_O_3_ layer, on the contrary, the largest drop in the response is observed. It can be assumed that, when doping with Fe_2_O_3_, the observed phenomena are related to the participation of iron cations directly on the surface of the ZnO film in the detection mechanism. Iron in oxides is known in two stable oxidation degrees: +2 and +3, which correspond to the oxides FeO, Fe_2_O_3_, and Fe_3_O_4_. After sorption of water molecules, we can assume the following reactions of iron cations with hydroxyl groups:

Fe^2+^ + OH^−^ → Fe^3+^ + H_2_O(g) + O^−^(ads) + e^−^ (3)

Fe^3+^ + e^−^ → Fe^2+^ (4)

Thus, a small iron content contributes to leveling the influence of moisture. With further increase in the iron content, reactions (3–4) begin to compete with those resulting in a response to NO_2_, which leads to an increase in the response drop. Nevertheless, this mechanism has not been experimentally confirmed and requires further study.

#### 3.4.3. ZnO/Fe_2_O_3_ Nanostructured Thin Films As Gas Biosensors

The obtained ZnO/Fe_2_O_3_ thin-film nanocomposites showed extremely high sensitivity to ultra-low NO_2_ concentrations (300–2000 ppb). Nitrogen dioxide is a gas biomarker for diseases associated with inflammatory processes in the lungs: bronchial asthma, bronchiectasis, and chronic obstructive pulmonary disease (COPD) [32,36,37]. The concentration of NOx in the exhaled air of a sick person, compared to the exhaled air of a healthy person, differs at the level of tens of ppb. Thus, the detection limit of NO_2_ by the ZnO/Fe_2_O_3_ thin-film nanocomposites obtained in the present work is not only much lower than the TLV, which is important in the context of environmental safety, but is also in a suitable range for medical purposes. Therefore, ZnO/Fe_2_O_3_ nanocomposites can be effective receptor materials in the composition of analytical devices for noninvasive diagnosis of diseases related to lung disease.

**Figure 14:**
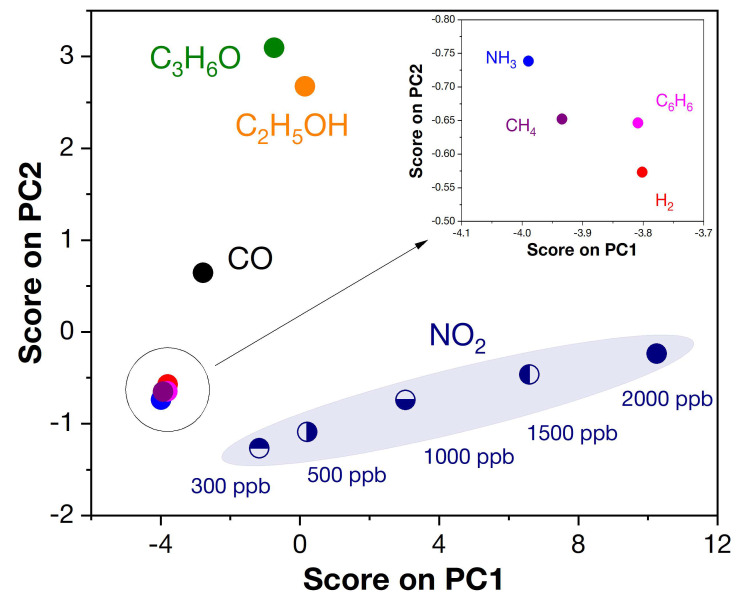
Pattern recognition based on PCA analysis for ZnO/Fe_2_O_3_ thin film nanocomposites.

Additionally, to enable selective signal separation, we used principal component analysis (PCA) to evaluate the correlation between the response of the four receptor nanomaterials used (Z, Z1Fe, Z2Fe and Z3Fe) and selectivity. Chemometric analysis is quite common in multisensor and E-nose systems for the detection of a wide group of analyte gases, including medical applications. Figure 14 shows the data obtained after mathematical processing of the PCA responses using the covariance matrix. As can be seen, the resulting graph consists of several distant clusters: 300–2000 ppb NO_2_, 100 ppm C_2_H_5_OH, C_3_H_6_O, and CO, and 1000 ppm CH_4_, H2, 100 ppm C_6_H_6_, and NH_3_ (inset), which allows their concentrations to be determined with high accuracy. Thus, using a sensor array consisting of four thin-film ZnO/Fe_2_O_3_ nanocomposites, signals can be separated with great selectivity when detecting the eight diverse analyte gases used in this work. Such results are extremely important in the transition to multisensor devices that are used for noninvasive disease diagnosis, where it is necessary to analyze a wide group of gases contained in human exhaled air.

Table 2 compares some gas-sensitive characteristics of ZnO/Fe_2_O_3_ nanocomposites with analogues presented in the literature. It is worth noting that the ZnO/Fe_2_O_3_ thin films obtained in our work have an improved set of gas-sensing properties: moderately low detection temperature, low detection limit, high response, and good selectivity.

## 4. Conclusions

ZnO/α-Fe_2_O_3_ nanocomposites were prepared by two-step aerosol vapor deposition (AACVD). Zinc and iron acetylacetonates in methanol were used as precursors, and synthesis was carried out at 400 °C with sequential sputtering of ZnO (60 min) and Fe_2_O_3_ (10, 20, 30 min). The iron content of the films with sputtering times of 10, 20, and 30 min was found to be 1.3, 4.8, and 5.8 at%, respectively, according to EDX data. The phase composition was determined by Raman spectroscopy, XPS and XRD were used for analysis, and the morphology was determined by SEM. ZnO particles have a wurtzite structure with an average size of 51–66 nm, and iron oxide particles with hematite structure on the surface of ZnO have an average size of 23–28 nm. All films have a large transmittance in the visible range. The optical band gap calculated by the Tauc method decreases with increasing Fe_2_O_3_ concentration from 3.26 to 3.16 eV. The films obtained showed a high response to 100 ppm acetone and ethanol at 225–300 °C and a high and selective response to 300–2000 ppb NO_2_ at 175 °C. The effect of humidity on the magnitude and shape of the signal obtained in NO_2_ detection was studied. The sensor with 1.3 at% Fe has the highest response, selectivity coefficient, and it has lower dependence on humidity, which can be explained by the formation of a heterojunction at the boundary of the two semiconductors.

## Figures and Tables

**Table 1:** Various characteristics of the obtained ZnO/Fe_2_O_3_ nanostructured thin films.

Sample	Content Fe, at.%	Average Crystallite Size (L), nm	Average Particle Size of ZnO, nm	Average Particle Size of Fe_2_O_3_, nm	Average Film Thickness, nm	Eg, eV	Response (S) to 300–2000 ppb NO_2_	Selectivity Coefficient (Sel)	Response Drop (RD) at 25–75%RH	Response Time, c
Z	0	40	66	-	174	3.26	1.3–4.0	1.2	11.1–15.6	143–180
Z1Fe	1.3	42	50	-	181	3.26	4.0–14.2	3.4	4.2–12.8	118–203
Z2Fe	4.8	30	51	23	185	3.23	1.9–6.1	1.7	9.3–21.9	121–160
Z3Fe	5.8	35	-	28	198	3.16	1.3–2.6	0.6	16.7–36.7	58–176

**Table 2:** Comparison of gas sensing properties of various ZnO-Fe_2_O_3_-based composites sensors in present work and literatures.

Composition	Synthesis/Coating Method	Gas	Conc, ppm	Temp, °C	Response	Response Time (s)	Selectivity	Ref.
α-Fe_2_O_3_-ZnO	Hydrothermal method	NO_2_	10	300	6.34	26	-	[67]
ZnO-Fe_2_O_3_	Co-precipitation method	NO_2_	250	400	10.53	1000	-	[68]
ZnFe_2_O_4_ nanosheets	Soaking, freeze-drying and calcination	Acetone	50	220	64.9	23	1.6	[69]
α- Fe_2_O_3_-ZnO core-shell nanowires	thermal oxidation/ ALD	H_2_S	5	250	5.98	81	5.2	[70]
Fe_2_O_3_-ZnO Nanograins	solid-state reaction method/RF magnetron sputtering	H_2_O_2_	1.5	RT + UV	12	91	4.9	[71]
Fe_2_O_3_-ZnO	solid-state reaction method/RF magnetron sputtering	NH_3_	548	250	6	~70	-	[72]
Fe_2_O_3_-ZnO	solid-state reaction method/RF magnetron sputtering	H_2_	2000	100	5045	372	>40	[73]
ZnO/α-Fe_2_O_3_ core-shell nanorods	chemical solution method/ionic-layer adsorption and reaction method	Ethanol	400	240	39	8	~4.9	[1]
ZnO-Fe_2_O_3_	solvothermal method	Acetone	100	290	29.9	-	1.4	[24]
ZnO-Fe_2_O_3_	thermal evaporation/solvothermal deposition	Ethanol	2000	200	~70	~115	-	[23]
tetrapod ZnO doped with Fe_2_O_3_	flame transport synthesis/ annealing with Fe	Ethanol	100	RT	51	2.5	20.4	[25]
ZnO-Fe_2_O_3_	vapor–liquid–solid process/sol–gel process	CO	100	300	18.8	~200	3	[26]
ZnO-Fe_2_O_3_	AACVD	NO_2_	2	175	14.2	118	3.4	This work

## Data Availability

Not applicable.
